# Cushing’s syndrome is associated with altered adipokine profile

**DOI:** 10.3389/fendo.2022.1032329

**Published:** 2022-12-06

**Authors:** Daniela Dadej, Ewelina Szczepanek-Parulska, Elżbieta Wrotkowska, Marek Ruchała

**Affiliations:** ^1^ Department of Endocrinology, Metabolism and Internal Medicine, Poznan University of Medical Sciences, Poznan, Poland; ^2^ Doctoral School, Poznan University of Medical Sciences, Poznan, Poland

**Keywords:** adipose endocrine, fetuin A, fatty acid-binding protein 4, retinol binding protein 4, cushing’s syndrome, integrative endocrine

## Abstract

**Introduction:**

Adipokines are signaling molecules involved in the integration of metabolism. Changes in their concentrations were observed in obesity, metabolic syndrome, diabetes mellitus and cardiovascular diseases, as well as endocrine disorders. Cushing’s syndrome is associated with metabolic dysregulation, but the significance of adipokines in this entity and related complications is largely unknown. The aim of our study was to determine the concentrations of adipokines: fetuin A, fatty acid binding protein 4 (FABP4) and retinol binding protein 4 (RBP4) in Cushing’s syndrome and to assess their relation to established cardiovascular and diabetes risk markers.

**Methods:**

We examined 21 subjects with Cushing’s syndrome and 24 healthy controls in a cross-sectional manner. Venous blood samples were analysed for adipokines, cortisol, adrenocorticotrophin, glucose, insulin, glycated haemoglobin (HbA1c), triglycerides, cholesterol fractions, thyrotropin and free thyroid hormones concentrations. Patients’ body mass index (BMI) was evaluated, homeostatic model assessment-insulin resistance and Systematic Coronary Risk Evaluation (SCORE) were calculated.

**Results:**

We found that the concentration of fetuin A was lower, while FABP4 and RBP4 concentrations were higher in Cushing’s syndrome compared to controls [156.4 ± 60.0 µg/ml vs 260.7 ± 49.6 µg/ml; 79.8 (35.2-156.1) ng/ml vs 27.9 (17.1-36.7) ng/ml and 34 (30-37.7) mg/l vs 25.8 (23.6-27.7) mg/l, respectively]. Fetuin A correlated inversely, while FABP4 and RBP4 positively, with the concentrations of urinary free cortisol and adrenocorticotrophin. Fetuin A was positively related to LDL-cholesterol, and negatively to SCORE and HbA1c. FABP4 was associated positively with BMI, HbA1c and triglycerides, while RBP4 correlated positively with triglycerides and systolic blood pressure.

**Conclusions:**

Adipokines’ concentrations change in hypercortisolism. Further research is needed to ascertain whether adipokines are involved in the development of metabolic complications accompanying Cushing’s syndrome or secondarily reflect metabolic dysregulation.

## Introduction

Adipokines are the cytokines secreted from adipocytes, that participate in signalling between tissues involved in the regulation of body metabolism. Altered adipokine production reflects adipose tissue dysfunction, that has been linked to obesity and associated complications (1). Recently, fetuin A, fatty acid binding protein 4 (FABP4) and retinol binding protein 4 (RBP4) were identified as biomarkers of atherosclerotic cardiovascular disease and diabetes (2–5). Several works demonstrated an association between these adipokines and endocrine disorders related to metabolic diseases (6, 7). Cushing’s syndrome (CS) has a well-established association with central adiposity, insulin resistance, hyperglycaemia and diabetes, as well as cardiovascular complications, that account for commonest causes of death (8–12). The risk of complications increases with disease duration and often persists despite biochemical control is attained. The early recognition of comorbidities and treatment optimisation are essential to achieve favourable outcome. Limited research regarding FABP4 in CS exists (13), while fetuin A and RBP4 have not been evaluated yet.

The aim of this study was to determine whether hypercortisolism affects the concentrations of fetuin A, FABP4 and RBP4 and to assess their association with established biomarkers of cardiovascular disease and diabetes, including lipid profile, glycated haemoglobin, homeostatic model assessment-insulin resistance (HOMA-IR) and Systematic Coronary Risk Evaluation (SCORE and SCORE2).

## Materials and methods

### Group characteristics:

The study participants were recruited between October 2019 and June 2022 at the Department of Endocrinology, Metabolism and Internal Medicine, Poznan University of Medical Sciences. The study group comprised 21 subjects newly diagnosed with endogenous CS in the course of Cushing’s disease (CD) (10 patients), ectopic ACTH production due to neuroendocrine tumours (5 patients) and small cell lung cancer (2 patients) or cortisol-secreting adrenal tumours (4 patients). The diagnosis of CS was made based on clinical features and laboratory findings according to clinical practice guidelines (14). The control group involved 24 individuals without severe chronic diseases, matched for age, gender, and BMI. The exclusion criteria for both groups were as follows: chronic liver or kidney disease, major cardiovascular events (myocardial infarction, stroke), diabetes, other uncontrolled endocrine disorders. Patients with subclinical CS were also excluded. Medication use was limited to antihypertensive agents (12 participants in the CS group and 2 in the control group), statins (2 CS subjects and 1 healthy control), metformin (3 subjects in the CS group), levothyroxine (6 participants in the control group and 4 in CS group), proton-pump inhibitors (3 individuals in the CS group and 2 in the control group), potassium and vitamin D supplementation.

### Study protocol:

All enrolled subjects underwent a full clinical examination. Blood samples were taken after an overnight fast. The following biochemical measurements were performed: creatinine, uric acid, fasting glucose, insulin, total cholesterol, LDL cholesterol, HDL cholesterol, triglycerides, thyroid stimulating hormone (TSH), free triiodothyronine (fT3), free thyroxine (fT4), dehydroepiandrosterone sulphate (DHEA-S), sex hormone binding globulin (SHBG), serum and urinary free cortisol (UFC) using Cobas 8000 modular analyser (Roche Diagnostics, Basel, Switzerland), glycated haemoglobin (HbA1c) using high performance liquid chromatography - D10 system (Bio-Rad Laboratories, California, USA). For statistical analyses, we used average UFC calculated from three consecutive measurements. The samples for determination of serum fetuin A, FABP4 and RBP4 were frozen in minus 80 degrees Celsius and stored. After recruitment completion, the analyses were performed using commercially available enzyme linked immunosorbent assay kits: Human fetuin A ELISA kit (BioVendor Laboratory Medicine Cat# RD191037100), Human adipocyte FABP ELISA kit (BioVendor Laboratory Medicine Cat# RD191036200R, RRID : AB_2813774), Human RBP4 ELISA kit (Immundiagnostik AG Cat# K 6110).

Cardiovascular risk was estimated using the SCORE system for Polish population (15), as well as recently updated model - SCORE2 (16). SCORE and SCORE2 were estimated using charts with the inclusion of following factors: sex, age, smoking status, systolic blood pressure and total cholesterol for SCORE and sex, smoking status, age, systolic blood pressure and non-HDL cholesterol for SCORE2. The charts allow the assessment of individuals aged between 40 and 70 years old. Younger participants were not involved in SCORE/SCORE2 estimation and associated analyses. HOMA-IR was calculated using the following formula (17):


$$HOMA-IR=\frac{fasting\;concentration\;of\;insulin\;\left(mIU/ml\right)\;*\;fasting\;concentration\;of\;glucose\;\left(mmol/l\right)}{22,5}$$

### Bioethical statement

Bioethics Committee of the Poznan University of Medical Sciences approved the project (Resolution no. 118/21). All participants gave written, informed consent to participate in the study. The project was conducted in accordance with the Declaration of Helsinki.

### Statistical analysis

Data are expressed as mean ± standard deviation or median [quartiles] as appropriate. Normality was verified with Shapiro-Wilk test, while equality of variances was analysed using Fisher-Snedecor test. For comparisons between groups either Student’s *t* test or Mann-Whitney *U* (exact) test were applied. For correlations Pearson product-moment correlation or Spearman’s rank-order correlation tests were used. A *P*-value<0.05 was considered statistically significant. The acquired data were analysed using PQStat Software (2022). PQStat v.1.8.4.136.

## Results

Clinical characteristics and laboratory results of the study groups are presented in the **Table 1**. Analysed groups did not differ in terms of age and BMI. Although hypertension was diagnosed in 57% of CS subjects and only 8% of healthy controls, SBP did not differ significantly between the groups. 38% of individuals with CS and 29% of the controls had prediabetes, while the remaining 62% of CS group and 71% of the control group had normal glucose tolerance. We identified no significant differences in fasting plasma glucose, insulin and HOMA-IR between the groups. Mean HbA1c was about 10% higher in CS subjects, but remained below diabetes cut-off value in all subjects. Considering the lipid panel, groups differed significantly in terms of triglycerides only (higher concentrations were observed in CS subjects). Apart from adrenal hormones, thyroid function was evaluated. TSH and fT3 concentrations were significantly higher in the control group. Median SHBG concentration was almost half lower in CS individuals compared with controls.

**Table 1:** Clinical characteristics and metabolic profile of patients with Cushing’s syndrome and controls.

	Cushing’s syndrome (*n*=21)	Controls (*n*=24)	P value
Gender (% M/F)	14/86	17/83	
Age [years]	42.8 ± 17.2	42.7 ± 12.3	ns
BMI [kg/m^2^]	26.7 ± 5.2	25.3 ± 3.9	ns
SBP [mmHg]	135 [128-150]	128.5 [123.8-136.3]	ns
Fasting glucose [mg/dl]	100 [86-115]	94.5 [87.8-100.3]	ns
Fasting glucose [mmol/l]	5.6 [4.8-6.4]	5.3 [4.9-5.6]	ns
Insulin [μU/ml]	13.4 [8.9-21.6]	12.2 [9.2-17.4]	ns
HOMA-IR	4.2 [1.9-5.1]	3.1 [2.1-4.3]	ns
HbA1c [%]	5.9 ± 0.4	5.3 ± 0.3	<0.001*
Total cholesterol [mg/dl]	196.3 ± 61.7	192.7 ± 34.0	ns
LDL cholesterol [mg/dl]	104.4 ± 47.8	115.5 ± 34.4	ns
HDL cholesterol [mg/dl]	51 [42-70]	66 [56.8-74.3]	ns
Non-HDL cholesterol [mg/dl]	132.7 ± 66.1	124.9 ± 35.6	ns
SCORE	1.5 [1-6.25]	1 [0.5-1.5]	ns
SCORE2	5 [2-9]	3 [2-5.5]	ns
Triglycerides [mg/dl]	157 [111-205]	110.5 [88-131.5]	0.008**
Creatinine [mg/dl]	0.73 [0.6-0.81]	0.76 [0.7-0.9]	ns
Uric acid [mg/dl]	4.9 [4.0-5.8]	4.1 [3.8-5.2]	ns
UFC [nmol/24h]	1370 [635.5-4567.7]	62.8 [55.3-88.3]	<0.001**
DHEA-S [μg/dl]	416.5 [183.8-582.5]	157 [109.5-223.3]	0.003**
SHBG [nmol/l]	27.8 [20.5-47.5]	53.7 [44.4-94.7]	0.001**
TSH [μU/ml]	0.8 [0.3-1.4]	1.5 [0.9-2.3]	0.011**
fT3 [pmol/l]	3.6 [2.5-4.1]	4.7 [4.4-5.4]	<0.001**
fT4 [pmol/l]	14.2 [13.2-17.3]	15.5 [14.2-16.9]	ns

Data are expressed as mean ± standard deviation when normally distributed and median [quartiles] when non-normally distributed.

*Student’s t test; **Mann-Whitney U test.

BMI, body mass index; SBP, systolic blood pressure; HOMA-IR, Homeostatic Model Assessment – Insulin Resistance; HbA1c, glycated haemoglobin; LDL, low density lipoprotein; HDL, high density lipoprotein; SCORE, Systematic Coronary Risk Estimation; UFC, urinary free cortisol; DHEA-S, dehydroepiandrosterone sulphate; SHBG, sex hormone binding globulin; TSH, thyroid stimulating hormone; fT3, free triiodothyronine; fT4, free thyroxine; ns, non-significant.

**Figure 1:**
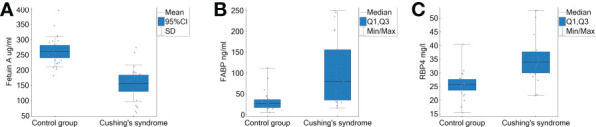
Serum concentrations of fetuin A, FABP4 and RBP4 in patients with Cushing’s syndrome and controls. **(A)** Fetuin A; Student’s *t* test p<0.000001. **(B)** FABP4; Mann-Whitney *U* test p between 0.0001 and 0.001. **(C)** RBP4; Mann-Whitney *U* test p between 0.0001 and 0.001. FABP4, fatty acid binding protein 4; RBP4, retinol binding protein 4; UFC, urinary free cortisol.

**Figure 2:**
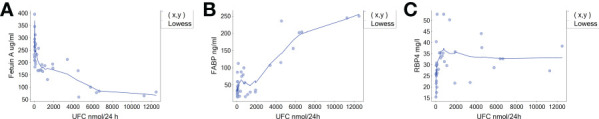
Correlations between adipokines and 24-hour urinary free cortisol. **(A)** Fetuin A and UFC. **(B)** FABP4 and UFC. **(C)** RBP4 and UFC. Spearman’s rank order correlation test; Lowess, locally weighted scatterplot smoothing; FABP4, fatty acid binding protein 4; RBP4, retinol binding protein 4; UFC, urinary free cortisol.

The comparison of the analysed adipokines’ serum concentrations between the study groups is shown in the **Figure 1**. Patients with CS presented significantly lower fetuin-A concentration and higher circulating FABP4 and RBP4 compared with healthy controls [156.4 ± 60.0 µg/ml vs 260.7 ± 49.6 µg/ml; 79.8 (35.2-156.1) ng/ml vs 27.9 (17.1-36.7) ng/ml and 34 (30-37.7) mg/l vs 25.8 (23.6-27.7) mg/l, respectively]. The adipokines’ concentrations correlated with 24-hour UFC: fetuin A negatively (*r*=-0.810, *p*=<0.001), while FABP4 (*r*=0.560, *p*=0.001) and RBP4 (*r*=0.489, *p*=0.002) positively, as shown in the **Figure 2**. We observed an inverse correlation between fetuin A and ACTH and a positive correlation of FABP4 and RBP4 with ACTH, after exclusion of subjects with adrenal CS; data presented in the **Table 2**.

**Table 2:** Correlation between adipokines and ACTH (n=41).

	Fetuin A		FABP4		RBP4	
	*r*	*p*	*r*	*p*	*r*	*p*
ACTH	-0.646	<0.001	0.483	0.001	0.382	0.014

r, Spearman’s rank order correlation coefficient; ACTH, adrenocorticotropic hormone; FABP4, fatty acid binding protein 4; RBP4, retinol binding protein 4.

Associations between fetuin A, FABP4, RBP4, metabolic and hormonal parameters are summarised in the **Table 3**. Only RBP4 was associated with SBP. On the other hand, the comparison between normotensive and hypertensive CS subjects revealed significant differences solely in fetuin A concentrations, which were significantly lower in hypertensive compared to normotensive CS patients (126.558 ± 54.556 µg/ml vs 196.278 ± 42.247 µg/ml, respectively; *p*= 0.005). We found no correlations between adipokines and fasting glucose or HOMA-IR. Moreover, adipokines’ concentrations did not differ significantly between CS subjects with normal glucose tolerance and prediabetes. Fetuin A correlated negatively with HbA1c, while FABP4 - positively. The lipid panel measurements were not related to adipokines’ concentrations, with the exception of LDL cholesterol, which correlated positively with fetuin A and triglycerides, which were positively related to FABP4 and RBP4. Only fetuin A correlated with SCORE. Fetuin A correlated positively with TSH, fT3, fT4 and SHBG. Conversely, FABP4 and RBP4 correlated negatively with TSH, thyroid hormones and SHBG.

**Table 3:** Correlation between adipokines and clinical variables (n=45).

	Fetuin A		FABP4		RBP4	
	*r ^a or b^*	*p*	*r ^b^*	*p*	*r ^b^*	*p*
Age	-0.277 ^a^	ns	0.204	ns	0.096	ns
BMI	0.009 ^a^	ns	0.460	0.002	0.095	ns
SBP	-0.205 ^b^	ns	0.266	ns	0.373	0.012
Fasting glucose	-0.009 ^b^	ns	0.261	ns	0.170	ns
HOMA-IR	0.139 ^b^	ns	0.114	ns	0.009	ns
HbA1c	-0.449 ^a^	0.006	0.622	<0.001	0.297	ns
Total cholesterol	0.083 ^a^	ns	-0.136	ns	0.062	ns
LDL cholesterol	0.333 ^a^	0.038	-0.314	ns	-0.062	ns
Non-HDL cholesterol	-0.004 ^a^	ns	-0.143	ns	0.108	ns
HDL cholesterol	0.184 ^b^	ns	-0.208	ns	-0.209	ns
Triglycerides	-0.143 ^b^	ns	0.339	0.030	0.470	0.002
Uric acid	-0.036 ^b^	ns	0.226	ns	0.384	ns
SCORE	-0.535 ^b^	0.009	0.162	ns	0.253	ns
SCORE2	-0.302 ^b^	ns	0.216	ns	0.322	ns
DHEA-S	-0.405 ^b^	0.008	0.251	ns	0.335	0.030
SHBG	0.550 ^b^	<0.001	-0.387	0.014	-0.357	0.024
TSH	0.472 ^b^	0.001	-0.90	0.008	-0.333	0.025
fT3	0.708 ^b^	<0.001	-0.484	0.001	-0.433	0.003
fT4	0.306 ^b^	0.041	-0.158	ns	0.022	ns

r ^a^, Pearson product-moment correlation coefficient; r ^b^, Spearman’s rank order correlation coefficient; FABP4, fatty acid binding protein 4; RBP4, retinol binding protein 4; BMI, body mass index; SBP, systolic blood pressure; HOMA-IR, Homeostatic Model Assessment – Insulin Resistance; HbA1c, glycated haemoglobin; LDL, low density lipoprotein; HDL, high density lipoprotein; SCORE, Systematic Coronary Risk Estimation; DHEA-S, dehydroepiandrosterone sulphate; SHBG, sex hormone binding globulin; TSH, thyroid stimulating hormone; fT3, free triiodothyronine; fT4, free thyroxine; ns, non-significant.

**Figure 3:**
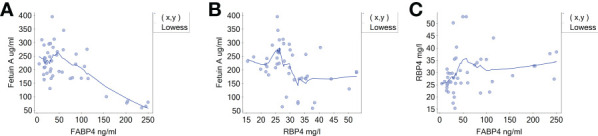
Correlations between adipokines. **(A)** Fetuin A and FABP4. **(B)** Fetuin A and RBP4. **(C)** RBP4 and FABP4. Spearman’s rank order correlation test; Lowess, locally weighted scatterplot smoothing; FABP4, fatty acid binding protein 4; RBP4, retinol binding protein 4.

Adipokines were also related to each other (**Figure 3**). We found an inverse correlation between fetuin A and FAB4 (r=-0.387, p=0.009), fetuin A and RBP4 (r=-0.421, p=0.004), while FABP4 and RBP4 (r=0.416; p=0.004) were positively related.

## Discussion

In this study, we found that fetuin A concentration is significantly lower, while FABP4 and RBP4 concentrations are significantly higher in CS compared to healthy controls. Adipokines correlated with 24-hour UFC and ACTH.

This is the first study to evaluate fetuin A and RBP4 concentrations in CS, which makes comparisons with the literature data challenging. Previously, Lewandowski et al. investigated the influence of two-day oral administration of dexamethasone on circulating RBP4 in healthy subjects and observed no change in RBP4 during the trial (18). Their results suggest, that RBP4 is not involved in short-term regulation of glucocorticoid-promoted effects. However, prolonged exposure to hypercortisolaemia, as observed in our study, may induce an increase in RBP4 either as a direct effect of cortisol excess or secondary to CS associated complications. With regard to FABP4, previous studies demonstrated its increased concentrations in patients with CS compared with lean, but not obese subjects (13), as well as a decline in FABP4 upon UFC normalisation with twelve months of pharmacological treatment (19). These studies identified no correlations between FABP4 and either plasma cortisol (13), or UFC (19). However, FABP4 was positively related to ACTH (19). Guarnotta et al. investigated patients with mild CD, with UFC about three times lower compared to our cohort (19). We suppose that the differences in magnitude of glucocorticoid excess and sample size contributed to the dissimilarities in obtained results.

Atherosclerotic cardiovascular complications and thromboembolic events are the commonest causes of death in patients with CS, accounting for almost 45% of mortality (9). Underlying mechanisms involve hypercoagulability and endothelial dysfunction due to hypercortisolaemia and associated comorbidities including hypertension, diabetes, dyslipidaemia etc., still, they are only partially understood (20). Fetuin A is involved in the regulation of calcium homeostasis as it binds surplus calcium, increases its clearance and prevents accumulation in vascular smooth muscle cells, thereby preventing promotion of coronary artery calcification and atherosclerosis (21, 22). Inverse relation between fetuin A and arterial calcification (23), as well as coronary artery disease (24), and cardiovascular mortality (4, 25), was reported. Increased Agatson score, a measure of coronary calcification, was observed in subjects with active CS or a history of CS (26), as well as a relation between increased cortisol reactivity to stress and greater extent of coronary artery calcification in short-time and prolonged observation – in healthy subjects (27, 28). Patients with CS have also increased concentrations of osteoprotegerin (29), a glycoprotein associated with coronary artery calcification (30). Whether low fetuin A in the course of CS, as observed in our study, contributes to increased coronary artery calcification and consequently cardiovascular mortality remains to be clarified. In line with this hypothesis, we observed an inverse correlation between fetuin A and SCORE, a surrogate for risk of fatal cardiovascular events.

Both FABP4 and RBP4 promote atherosclerosis *via* inducing endothelial dysfunction and foam cell formation (31, 32), and correlate with cardiovascular events (2, 33), though conflicting evidence exists (34). In obese non-diabetic patients RBP4 was associated with main features of atherogenic dyslipidaemia – low HDL cholesterol and high triglycerides (35, 36). We found a positive relation between RBP4 and triglycerides, that were significantly higher in CS group, and no correlation with HDL or other lipid panel measurements. We identified a positive correlation of RBP4 with systolic blood pressure, which predisposes to endothelial dysfunction as well. Data on dyslipidaemia in CS is limited. Usually raised triglycerides, total and LDL cholesterol, and reduced HDL cholesterol are observed (8, 37). However, abnormal lipid panel might result from obesity alone (38). Previous studies in subjects with CS have shown a positive correlation between FABP4 and triglycerides, and BMI (13, 19), as observed in our cohort, while correlations with cholesterol fractions were inconclusive. Whether adipokines may induce lipid profile abnormalities in CS, must be further investigated.

Glucose intolerance and diabetes are further conditions that commonly complicate CS (8). Glucocorticoids impair insulin sensitivity in muscles, adipose tissue and liver (39). At the same time they increase glucagon secretion, which stimulates gluconeogenesis and inhibits glycolysis. Analysed adipokines also contribute to the development of insulin resistance and diabetes. They disrupt insulin signalling in peripheral tissues, supressing glucose uptake and utilization (31, 40–42), and promote adipose tissue inflammation, and lipid induced insulin resistance (43–45). Recent study indicates, that FABP4 targets pancreatic β-cells directly and impairs glucose-stimulated insulin secretion (46). Similarly to previous studies in subjects with CS, we identified a positive correlation between FABP4 and HbA1c (13, 19). We detected no correlations between FABP4 and fasting plasma glucose, insulin or HOMA-IR. Previous results regarding HOMA-IR, insulin and fasting glucose were as well inconclusive (13, 19). Conversely to studies in normocortisolaemic individuals, we did not observe associations between fetuin A and HOMA-IR and found an inverse correlation with HbA1c. We can only speculate on the cause, as no study assessing interactions between glucocorticoids and fetuin A exists. Perhaps fetuin A, derived predominantly from the liver, is downregulated in CS due to increased protein oxidation and reduced protein synthesis (47). We identified no associations between RBP4 and carbohydrate homeostasis parameters in CS, opposed to most studies in normocortisolaemic subjects (6). Previous research indicates that RBP4 may be involved in the pathogenesis of insulin resistance and diabetes, however conflicting results have also been published. Several studies either failed to identify relations between glucose stimulated insulin secretion and RBP4 in diabetic and obese subjects or indicated that increase in RBP4 observed in glucose intolerant subjects is rather secondary and has no causal relationship (48, 49). High circulating RBP4 in CS may reflect patients’ metabolic state, but is unlikely to have a causative association.

Glucocorticoids have a well-known suppressive effect on TSH and thyroid hormones. Indeed, we observed significantly lower TSH and fT3 in CS group compared with controls. Recent studies indicate that adipokines are associated with thyroid status and may reflect or contribute to metabolic dysregulation accompanying thyroid dysfunction. Fetuin A was found to increase in hyperthyroidism, while results in hypothyroidism are inconclusive. Increased FABP4 concentrations were observed in both hypothyroid and hyperthyroid individuals as well as in autoimmune thyroiditis. RBP4 tends to increase in hypothyroidism. Adipokines’ concentrations were found to correlate mostly with TSH, but several studies revealed also associations with free thyroid hormones (6, 50). In line with previous study in CS subjects, we found a negative correlation between FABP4 and fT3 (13). The significance of this finding is unclear.

This study has some limitations. Firstly, the observational, cross-sectional study design precludes causal inferences. Secondly, the sample size is relatively small, which limited the application of statistical methods, including regression analyses and resulted in poor control of confounding factors, such as age, BMI, lipid profile or glucose. Thirdly, the influence of CS type on the obtained results cannot be excluded. Endogenous CS is a heterogeneous condition, with different course of the disease and prognosis depending on its aetiology and severity. Therefore, adipokines’ profile may as well differ between CS subpopulations. Insufficient number of participants in each CS type prevented us from analysing this issue. Finally, lack of prior research in the topic is both study strength and downside as it restricted the comparisons of results. Nonetheless, given the rarity of endogenous CS our results offer new observations in this population.

## Conclusions

We found significant alterations in adipokines’ concentrations in subjects with Cushing’s syndrome, that correlated with UFC and ACTH concentrations and selected metabolic parameters. Whether fetuin A, FABP4 and RBP4 participate in the development of metabolic complications accompanying CS or reflect metabolic dysregulation requires further investigation. Although UFC concentration and successful treatment determine the patient’s outcome, cortisol level alone is not sufficient to assess the risk of complications. Adipokines which mark the risk of metabolic complications might add novel information to prediction models. Defining the role of adipokines presents as a promising direction for further improvement of prevention and treatment of cardiovascular disease and diabetes in patients with CS.

## Data availability statement

The raw data supporting the conclusions of this article will be made available by the authors, without undue reservation.

## Ethics statement

This study was reviewed and approved by Bioethics Committee of the Poznan University of Medical Sciences, Poznan, Poland (Resolution no. 118/21). The participants provided their written informed consent to participate in this study.

## Author contributions

DD, ES-P and MR contributed to conception and design of the study. DD acquired, analyzed and interpreted the patient data and was a major contributor in writing the manuscript. EW performed the ELISA determinations of analyzed adipokines. ES-P and MR revised the manuscript. All authors contributed to the article and approved the submitted version.
